# The profile of genome-wide DNA methylation, transcriptome, and proteome in streptomycin-resistant *Mycobacterium tuberculosis*

**DOI:** 10.1371/journal.pone.0297477

**Published:** 2024-01-29

**Authors:** Zhuhua Wu, Haicheng Li, Jiawen Wu, Xiaoyu Lai, Shanshan Huang, Meiling Yu, Qinghua Liao, Chenchen Zhang, Lin Zhou, Xunxun Chen, Huixin Guo, Liang Chen

**Affiliations:** 1 Center for Tuberculosis Control of Guangdong Province, Guangzhou, China; 2 The Third Affiliated Hospital, Sun Yat-Sen University, Guangzhou, China; 3 Institute for tuberculosis control of Zhaoqing, Zhaoqing, China; Lady Hardinge Medical College, INDIA

## Abstract

Streptomycin-resistant (SM-resistant) *Mycobacterium tuberculosis* (*M*. *tuberculosis*) is a major concern in tuberculosis (TB) treatment. However, the mechanisms underlying streptomycin resistance remain unclear. This study primarily aimed to perform preliminary screening of genes associated with streptomycin resistance through conjoint analysis of multiple genomics. Genome-wide methylation, transcriptome, and proteome analyses were used to elucidate the associations between specific genes and streptomycin resistance in *M*. *tuberculosis* H37Rv. Methylation analysis revealed that 188 genes were differentially methylated between the SM-resistant and normal groups, with 89 and 99 genes being hypermethylated and hypomethylated, respectively. Furthermore, functional analysis revealed that these 188 differentially methylated genes were enriched in 74 pathways, with most of them being enriched in metabolic pathways. Transcriptome analysis revealed that 516 genes were differentially expressed between the drug-resistant and normal groups, with 263 and 253 genes being significantly upregulated and downregulated, respectively. KEGG analysis indicated that these 516 genes were enriched in 79 pathways, with most of them being enriched in histidine metabolism. The methylation level was negatively related to mRNA abundance. Proteome analysis revealed 56 differentially expressed proteins, including 14 upregulated and 42 downregulated proteins. Moreover, three hub genes (*coaE*, *fadE5*, and *mprA*) were obtained using synthetic analysis. The findings of this study suggest that an integrated DNA methylation, transcriptome, and proteome analysis can provide important resources for epigenetic studies in SM-resistant *M*. *tuberculosis* H37Rv.

## Introduction

Tuberculosis (TB) is a global public health challenge. This disease affects multiple organs in the human body, with the lungs being the most infected organ (approximately 80%–90% of cases) [1]. Since 1945, the emergence and application of antituberculosis drugs has become a milestone in the treatment of TB [2]. However, the decreasing incidence of TB has reversed yearly [3]. Meanwhile, the emergence of multidrug-resistant TB has worsened the management of TB [4]. Lipid content of the cell wall of *Mycobacterium tuberculosis (M*. *tuberculosis)* is as high as 60%. The capsule outside the cell wall is acid resistant [5–7]. In 1998, the Pasteur Institute obtained the genome sequence of the *M*. *tuberculosis* standard strain H37Rv [8, 9]. Subsequently, genome sequences of other strains, including *Leprosy bacillus* [10], several clinical isolates of *M*. *tuberculosis* [11], *Mycobacterium bovis* [12], *Mycobacterium bovis* BCG [13], and *M*. *tuberculosis* H37Ra [14] have been obtained. These works have led to the research of TB at the molecular level.

Streptomycin is an aminocycloalcohol glycoside antibiotic and the earliest effective antituberculosis drug. This drug inhibits TB protein synthesis in the ribosomal 30S small subunit of *M*. *tuberculosis* [15]. Currently, the mechanism underlying streptomycin resistance in *M*. *tuberculosis* has not been fully elucidated. However, evidence has shown that mutations in ribosome target genes are the main contributors to streptomycin resistance in *M*. *tuberculosis*. The primary mutant genes are ribosomal S12 encoding the protein (rps L gene) and 16S rRNA encoding the protein (rrs gene) [16]. Meanwhile, a previous study suggested that the highly conserved 16S rRNA 530-loop 7-methylguanosine methyltransferase encoded by *gid B* possessed a G527 methylation deletion, which led to low-level drug resistance in *M*. *tuberculosis* [17]. Ramaswamy S *et al*. showed that mutations in *rps L* and *rrs* were present in 65%–75% of clinically isolated streptomycin-resistant (SM-resistant) *M*. *tuberculosis* [18]. However, the relationship between the methylation genes and streptomycin resistance in *M*. *tuberculosis* requires further research.

The primary objective of this study was to perform preliminary screening to identify genes associated with streptomycin resistance using conjoint analysis of multiple genomics. High-throughput sequencing and isobaric tags for relative and absolute quantification (iTRAQ) and high-performance liquid chromatography (HPLC) labeling were used to probe the methylome atlas, transcriptome atlas, and proteome of the SM-resistant H37Rv strain and *M*. *tuberculosis* H37Rv strain without streptomycin resistance. In addition, molecular regulatory networks based on hub genes were constructed. The results of this study can help investigate streptomycin resistance in *M*. *tuberculosis* H37Rv.

## Materials and methods

### Bacteria treatment

In this study, M. *tuberculosis* H37Rv strain without streptomycin resistance was obtained from the Sample Bank of the Reference Laboratory of Guangdong Province. This strain was cultured in Löwenstein–Jensen (LJ) medium at 37°C for 4 weeks. Then, an agglomerated monoclonal was picked, dispersed, diluted to 1 Mech turbidity, and cultivated in LJ media for further amplification; this was named primary generation 0 (G0). A previous study showed that the SM-resistant *M*. *tuberculosis* H37Rv strain can survive in 2.0 μg/mL streptomycin [19]. Consequently, streptomycin-resistant strains that satisfied the WHO criteria of surviving in the presence of 4.0 μg/ml streptomycin concentration were developed [20]. First, LJ medium containing streptomycin was prepared at concentrations of 2^−4^, 2^−3^, 2^−2^, 2^−1^, 2^0^, and 2^2^. Second, LJ medium with 2^−4^ streptomycin was added to primary G0 strains and cultured for 4 weeks at 37°C; this was named G1. G1 strains were cultivated in LJ medium with 2^−3^ streptomycin for 4 weeks and named G2. G2 strains were cultivated in LJ medium with 2^−2^ streptomycin for 4 weeks to develop G3 strains. G4 strains were generated by cultivating G3 in LJ medium with 2^−1^ streptomycin for 4 weeks. The G4 strains were continuously cultured in LJ medium with 2^0^ streptomycin for 4 weeks. Then, *M*. *tuberculosis* H37Rv strains with/without streptomycin resistance were cultured for another 3 months in LJ medium with 2^2^ streptomycin at 37°C. The bacteria (three biological replicates) isolated from these two groups were collected and stored at −80°C for genome-wide DNA methylation, transcriptome, and proteome analysis. Streptomycin was purchased from Sigma-Aldrich (St. Louis, MO, USA).

### Methylome

DNA in the drug-resistant and control groups was extracted using the Quick-DNA Fungal/Bacterial Kit (Zymo Research, USA) according to the detailed protocol provided by the manufacturer. Subsequently, EZ DNA Methylation-GoldTM Kit (ZYMO, USA) was used for performing bisulfite treatment based on the manufacturer’s protocol. Then, bisulfite-treated DNA was used for library preparation using the bisulfite Accel-NGS Methyl-Seq DNA Library Kit (SWIFT, USA). In brief, the entire experimental procedure includes denaturation (95°C for 2 minutes and on ice for 2 minutes), adaptase treatment (37°C for 15 min, 95°C for 2 minutes, and 4°C hold), extension (98°C for 1 minute, 62°C for 2 minutes, 65°C for 5 minutes, and 4°C hold), and ligation (25°C for 15 min). Subsequently, indexing PCR was conducted in a 50-μL reaction system, including 25 μL sample and 25 μL premixed indexing PCR Reaction Mix. The thermal conditions were set as follows: 98°C for 30 secs, followed by nine PCR cycles: 98°C for 10 seconds, 60°C for 30 secs, and 68°C for 60 secs. Finally, the reaction was maintained at 4°C. The sample was transferred to a 1.5-mL tube. Indexing PCR clean up was conducted using beads (Beckman, USA) and freshly prepared 80% ethanol. The prepared library was sequenced using the pair-end strategy on the Illumina HiSeq2500 platform. The sequencing data were then analyzed following the method described in a previous study with a few modifications [21]. In brief, sequencing data were filtered to remove low-quality data and obtain clean data. Next, 49-bp reads without adaptor sequences were mapped to the reference genome (H37Rv: ASM19595v2 https://www.ncbi.nlm.nih.gov/assembly/GCF_000195955.2/). Subsequently, DNA methylation strand specificity was validated using BGI SOAPaligner version 2.01 [22]. The parameter was set to two mismatches for successful mapping. From the read alignment coordinates, DNA methylation regions were retrieved and saved as sequences in the respective FASTA files (one file per sample). The median read coverage of each DNA methylation region was then calculated from the output of the mpileup base calling algorithm (samtools-1.2). Next, the cytosine information was used to determine the significance of DNA methylation differences between the two groups. In our study, a false discovery rate (FDR) correction was applied to multiple testing (at the 0.05 level). Meanwhile, paired t-test was used to screen for significant differences in DNA methylation between the two groups. *P*-values of <0.01 were considered significantly different. Moreover, the fold change (FC) was calculated as the ratio of the level of methylation genes in the streptomycin-treated group to the normal group. Annotation was performed using ANNOVAR (version: 2019-06-28).

### Transcriptome

The 50-ml culture medium was concentrated via centrifugation in each group. Fast RNA Pro Blue Kit (MP Biomedicals, USA) was used for total RNA extraction using the manufacturer’s protocol. RNA purity was determined via 2% agarose gel electrophoresis, and its concentration and quality were tested using NanoDrop ND1000 (Fisher Scientific, USA). Then, DNA contamination and ribosomal RNA were removed using DNase I (Epicenter, USA) and Epicenter Ribo-zero^™^ rRNA Removal Kit (Epicenter, USA), respectively, following the manufacturers’ instructions. An ultrasonic method was used for fragmentation. The first and second strands were synthesized using random primers and dUTP. The RNA library was constructed using the kit from NEB (NEBNext^®^ Ultra^™^ Directional RNA Library Prep Kit for Illumina), purified using 0.8X beads (Beckman, USA), and assessed using an Agilent Bioanalyzer 2100 system (Agilent, USA). After quality assessment, the purified libraries were sequenced using an Illumina HiSeq 4000 platform (Illumina, USA). Then, the sequencing data were analyzed using a previously described method [21]. In brief, reads obtained from the sequencing machines included raw reads containing adapters or low-quality bases that could affect the following analysis. Thus, to obtain high-quality clean reads, reads were further filtered by removing the following: 1) reads containing adapters; 2) reads containing >10% of unknown nucleotides (N); and 3) low-quality reads containing >50% of low-quality (Q-value ≤ 20) bases. Clean reads were further used for alignment and analysis. Clean reads of each sample were then mapped to the reference genome using hisat2 (version 2.1.0) to generate bam files. After aligning with the reference genome, the bam files were entered into HTSeq (version 0.6.1) to generate read count files. Then, DEseq (from R package) was used to read the count files for differential expression analysis. The P-value was calculated, subjected to multiple hypothesis testing and correction, and its threshold was determined by controlling FDR. In this study, FDR value of ≤0.001 and |log2Ratio| value of ≥1 (the ratio of the level of differential expression genes in the streptomycin-treated group to the normal group) were used as screening criteria for differential expression genes between the two groups. The heat map was drawn using heatmap in the R package (version 3.3.3). KOBAS 3.0 (the website link as follows: http://kobas.cbi.pku.edu.cn/) was used for KEGG pathway analysis.

### iTRAQ and HPLC

In this study, proteins from the SM-resistant and normal groups were isolated with a buffer consisting of 50 mM Tris-HCl, 150 mM NaCl, 1% SDS, 0.1% Trionx‐100, 1% SDC (pH 8.0), 100 μg/ml PMSF, and 1% acetone. Then, the 8plex iTRAQ kit (AB Sciex) was used for labeling, and Bradford assay was used for evaluating the total protein concentrations. Subsequently, trypsin (Promega, Madison, WI, USA) was added for protein digestion, followed by iTRAQ labeling. Then, Thermo Scientific Q‐Exactive Quadrupole–Orbitrap Mass Spectrometer and Thermo Dionex Ultimate 3000 RSLCnano System were used for HPLC analysis. The protein expression profile was analyzed using proteome discoverer v1.4 (Thermo Scientific) and proteinpilot^™^ v5.0 (AB Sciex). An AVG value (fold change) of ≥1.5 indicated upregulated protein expression, whereas AVG value of ≤0.67 indicated downregulated protein expression.

### Protein–protein interaction (PPI)

PPIs were studied using Search Tool for the Retrieval of Inter-acting Genes (STRING) v10.0 (interaction score > 0.4 [medium confidence] as the cutoff score; website link: https://string-db.org/). A P-value of <0.05 was considered statistically significant.

### Statistical analysis

According to a previous study, the relationship between the differential methylation level and gene levels was analyzed using Spearman’s correlation analysis [23]. A P-value of <0.05 was considered statistically significant.

## Results

### Differentially methylated genes between the drug-resistant and normal groups

**Fig 1:**
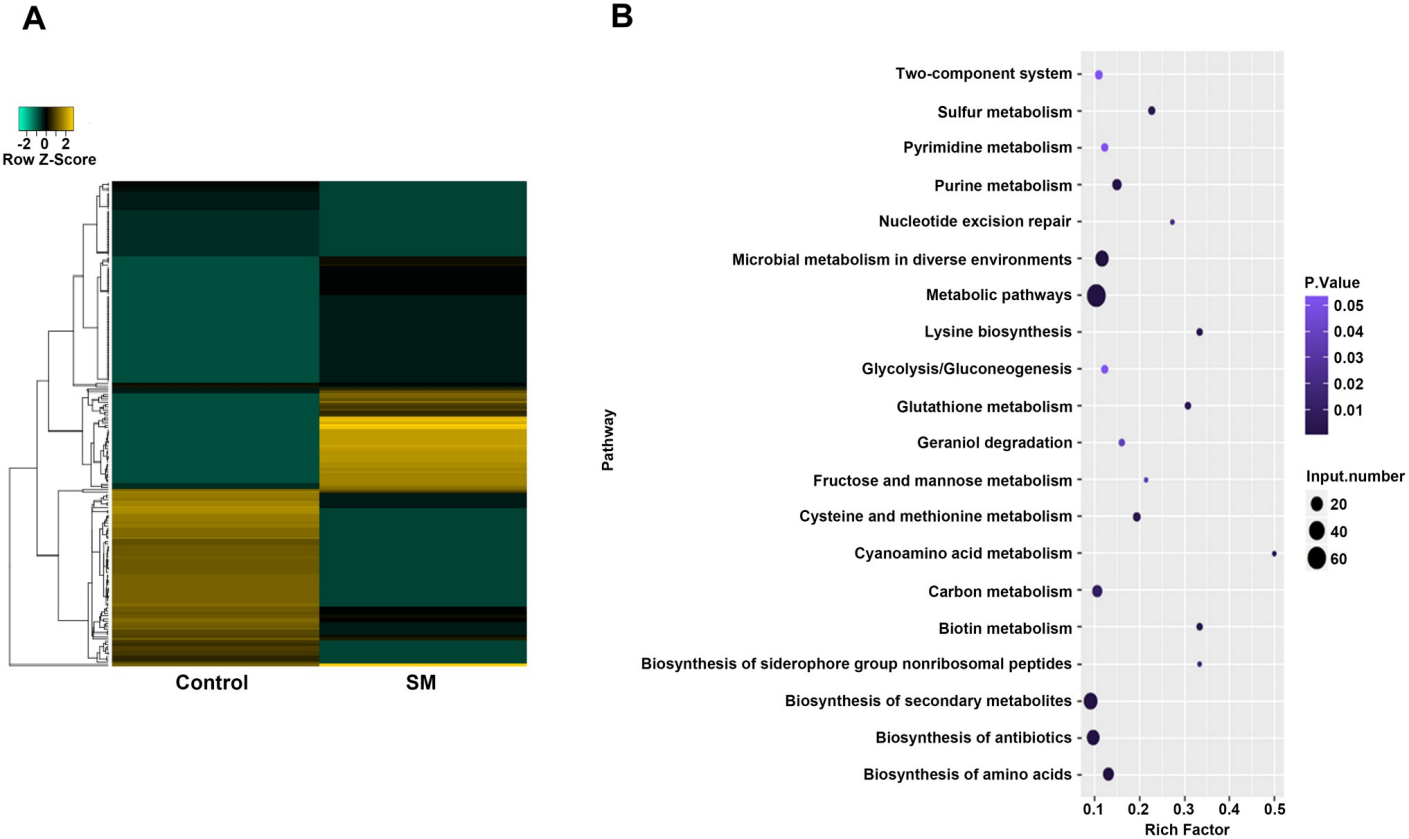
Differentially methylated genes in the streptomycin-resistant group and normal group were used for methylome atlas and KEGG analysis. A) The heat map was used to show the differential methylated genes in the streptomycin-resistant group compared with those in the normal group. B) KEGG analysis was used for comparing the differentially methylated genes in the streptomycin-resistant and normal groups. Rich factor indicated the ratio of the number of differentially methylated genes enriched in each KEGG term to the number of all annotated genes in the KEGG term. Negative binomial distribution model was used to calculate P-value.

In this study, differentially methylated genes were screened to investigate potential epigenetic changes. Fig 1A and S1 Table show that 188 genes were differentially methylated between the two groups. Of the 188 differentially methylated genes, 89 were hypermethylated in the drug-resistant group compared with those in the normal group. For example, *aceE* (FC = 8.00), *adoK* (FC = 2.00), and *argB* (FC = 3.00) were differentially hypermethylated genes. Meanwhile, 99 genes were hypomethylated in the drug-resistant group compared with those in the normal group. For example, *aceAa* (FC = 0.50), *adh* (FC = 0.02), and *alkA* (FC = 0.07) were the differential hypomethylated genes. KEGG analysis indicated that 188 differentially methylated genes were enriched in 74 pathways, and only 17 pathways showed significant P-values (Fig 1B and S2 Table). Metabolic pathways (mtu01100, corrected P-value = 1.28E-07) were the most enriched item. Sixty-two differentially methylated genes were enriched in this pathway, such that the genes were also enriched in microbial metabolism in diverse environments (mtu01120, corrected P-value = 1.87E-03) and amino acid biosynthesis (mtu01230, corrected P-value = 1.49E-02). Twenty-five differentially methylated genes were involved in microbial metabolism in diverse environments and 15 differential methylated genes were associated with the biosynthesis of amino acids.

### Differentially expressed genes between the drug-resistant and normal groups

**Fig 2:**
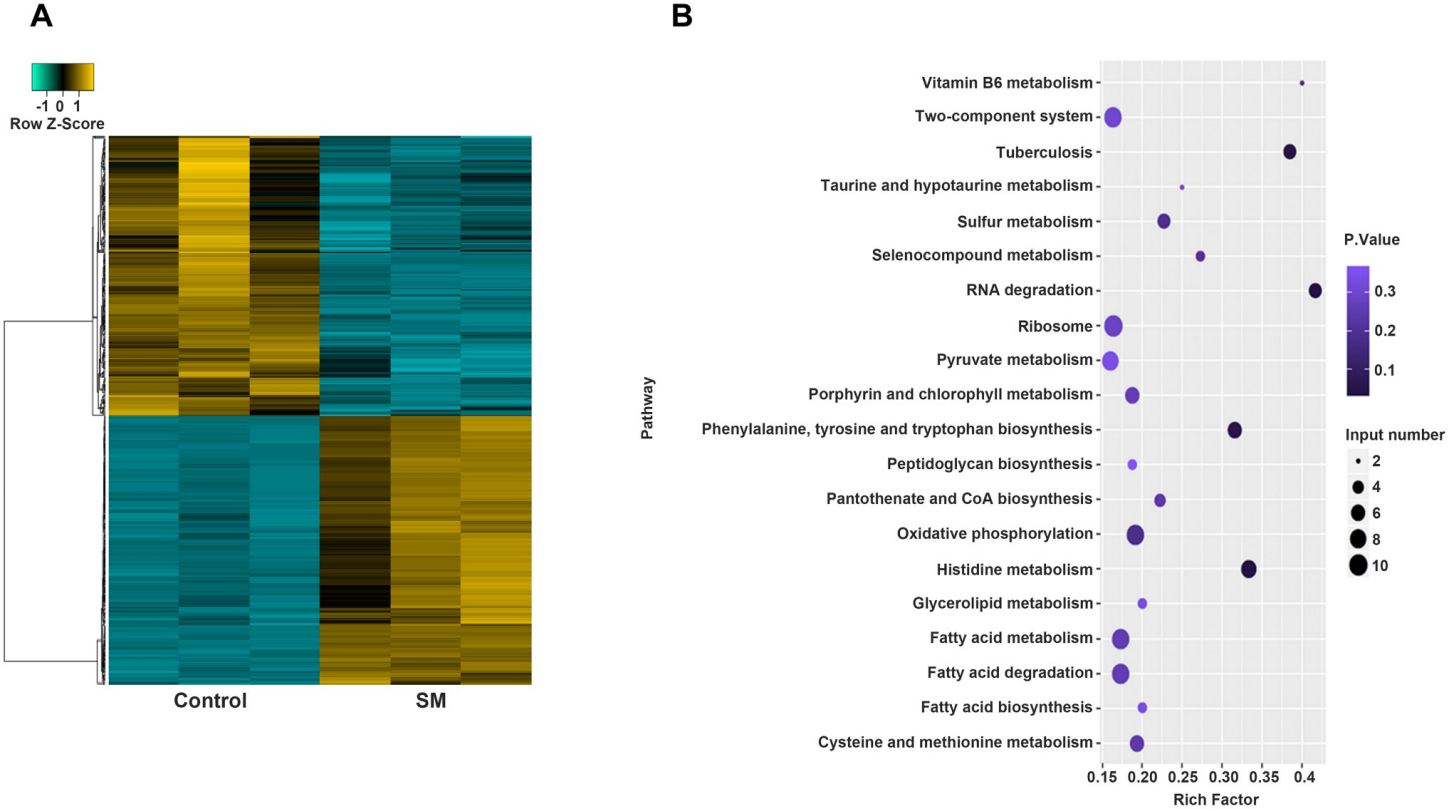
Transcriptome atlas and KEGG analysis of differentially expressed genes between the streptomycin-resistant and normal groups. A) The heatmap was used to show the differentially expressed genes between the streptomycin-resistant and normal groups. B) KEGG analysis was used to compare the differentially expressed genes in the streptomycin-resistant and normal groups. Rich factor indicated the ratio of the number of differentially expressed genes enriched in each KEGG term to the number of all annotated genes in the KEGG term. Negative binomial distribution model was used to calculate P-value.

To study potential changes in mRNA abundance, we used high-throughput RNA sequencing. Fig 2A and S3 Table show that 516 genes were differentially expressed between the drug-resistant and normal groups. Among the 516 genes, 263 genes, including *RVnc0036a* (FC = 0.447343), *Rv0115a* (FC = 0.114274), and *Rv3136A* (FC = 0.248208), were significantly more highly expressed in the drug-resistant group than in the normal group. Meanwhile, the expression of 253 genes, including *Rv2308a* (FC = 2.18E+00), *Rv0609A* (FC = 2.75E+00), and *Rv3294c* (FC = 2.68E+00), was significantly lower in the drug-resistant group than in the normal group. KEGG analysis indicated that the 516 differentially expressed genes were enriched in 79 pathways, and only 25 pathways showed significant P-values (Fig 2B and S4 Table). Most genes were enriched in histidine metabolism (mtu00340, P-value = 0.034). Seven differentially expressed genes were identified. Notably, these genes were also enriched in RNA degradation (mtu03018, P-value = 0.037) and tuberculosis (mtu05152, P-value = 0.046). Five differentially expressed genes from each pathway were retrieved.

### Spearman’s correlation analysis of DNA methylation and gene expression

**Fig 3:**
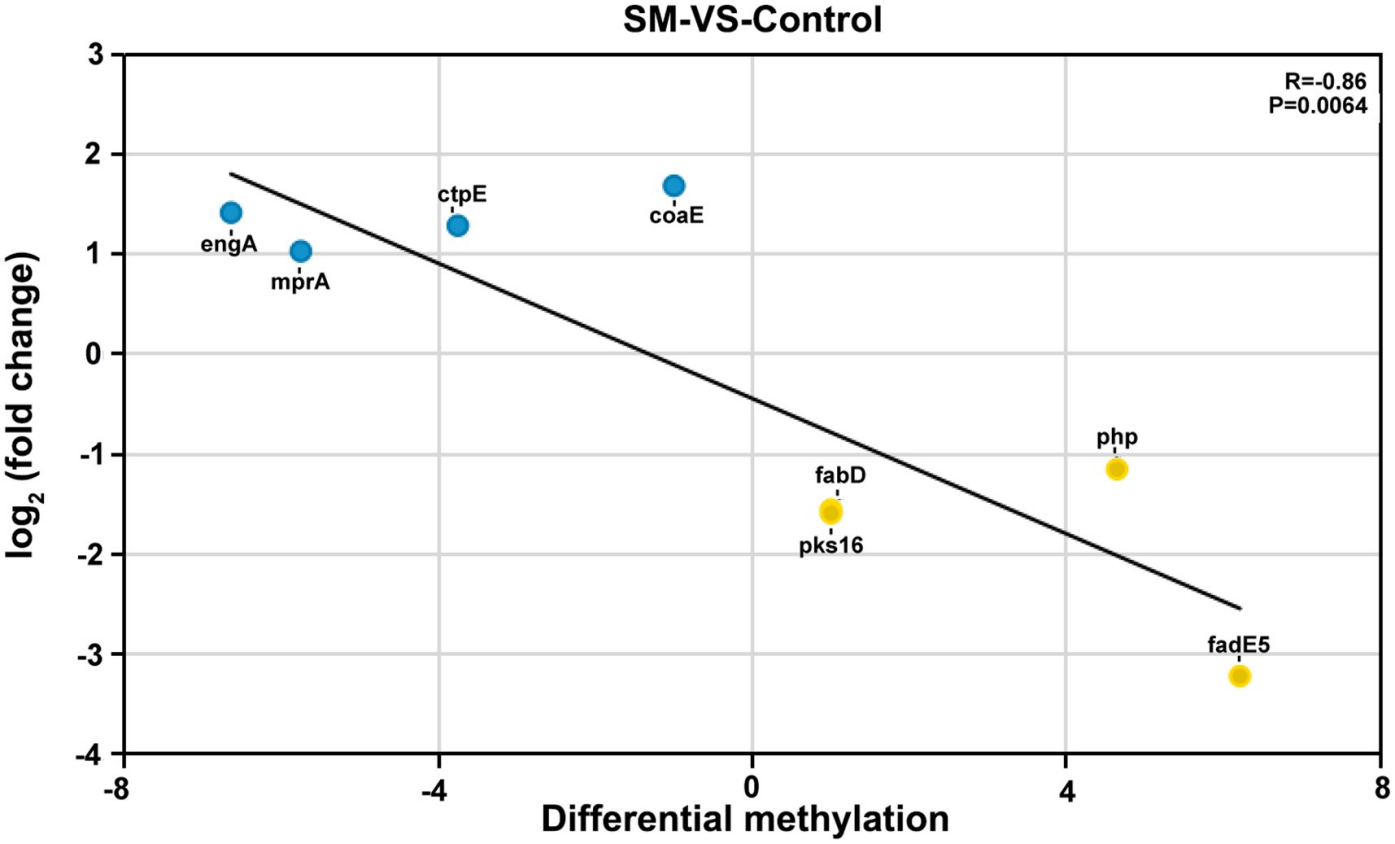
Spearman’s correlation analysis between the expression of five genes and their DNA methylation status. Scatterplots were used to show the gene expression with fold change vs. the change of DNA methylation in streptomycin-resistant *M*. *tuberculosis* H37Rv. SM: streptomycin-resistant group (drug-resistant group); Control: without streptomycin-treated group (normal group). The yellow dot indicates that the DNA methylation level was upregulated, whereas the gene expression level was downregulated; the blue dot indicates that the DNA methylation level was downregulated, whereas the gene expression level was upregulated. Spearman’s correlation analysis was used to calculate P-value.

Simultaneous changes in the methylation level and mRNA abundance can have significant biological implications. In this study, we compared the differentially expressed genes and differentially methylated genes harvested. Fig 3 shows that the methylation difference level was negatively related to mRNA abundance (correlation coefficient = −0.86, P = 0.0064), as determined using Spearman’s correlation analysis.

### PPI network analysis of hub genes from three omics

**Fig 4:**
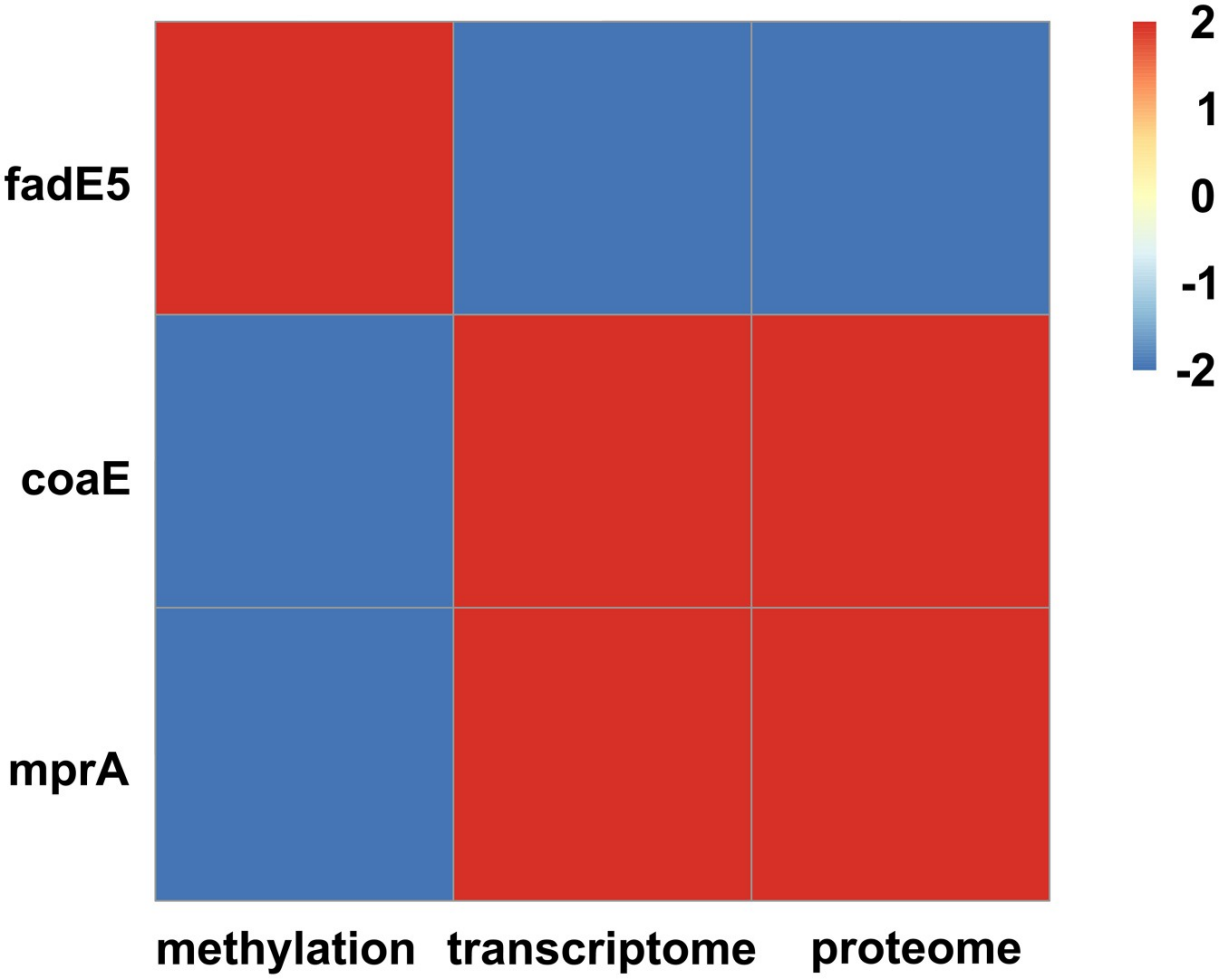
Correlation among the methylation, mRNA, and protein levels of *coaE*, *fadE5*, and *mprA*. The heat map was adopted to establish the expression correlation of hub genes from three omics in streptomycin-resistant *M*. *tuberculosis* H37Rv. Spearman’s correlation analysis was used to calculate P-value.

**Fig 5:**
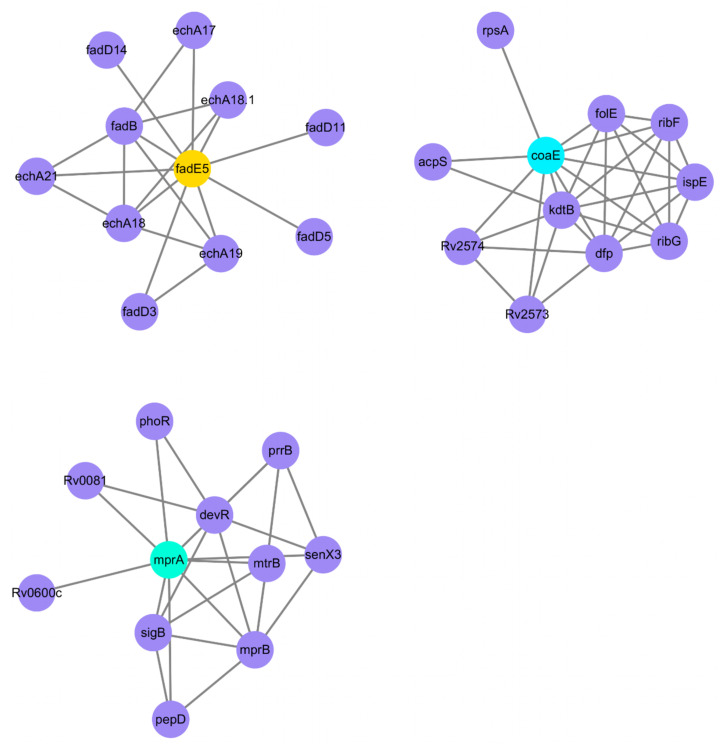
Protein–protein interaction network analysis of hub genes from three omics. The STRING 10.0 database was adopted to establish the PPI network of hub genes from three omics in streptomycin-resistant *M*. *tuberculosis* H37Rv. The yellow dot indicated that the DNA methylation level of *fadE5* was upregulated, the blue dot indicated that the DNA methylation level of *coaE* and *mprA* was downregulated, and the purple dot indicated associated proteins. The STRING database was used for PPI analysis.

Overall, 56 differentially expressed proteins (14 upregulated and 42 downregulated proteins) between the SM-resistant and normal groups were identified (S5 Table). Further analysis (https://bioinfogp.cnb.csic.es/tools/venny/index.html) of methylation, transcriptome, and proteome of SM-resistant *M*. *tuberculosis* led to the identification of three hub genes (*coaE (Dephospho-CoA kinase)*, *fadE5 (Probable acyl-CoA dehydrogenase FADE5)*, and *mprA (DNA-binding transcriptional repressor)*). The results showed that compared with the control group, the methylation levels of *coaE* and *mprA* were significantly downregulated and their transcription and protein levels were significantly upregulated, whereas the methylation levels of *fadE5* were significantly upregulated and their transcription and protein levels were significantly downregulated in *M*. *tuberculosis* with streptomycin resistance (Fig 4, S6 Table). In addition, we used the STRING 10.0 database to analyze the PPI networks of three hub genes (*coaE*, *fadE5*, and *mprA*) in SM-resistant *M*. *tuberculosis* H37Rv. Fig 5 and S7 Table show that *fadE5* was associated with *fadD14*, *fadD3*, *fadD5*, *fadD11*, *echA17*, *echA18*.*1*, *echA21*, *echA18*, *echA19*, and *fadB*; *coaE* was associated with *acpS*, *rpsA*, *folE*, *ribF*, *ispE*, *ribG*, *dfp*, *kdtB*, Rv2573, and Rv2574; and *mprA* was associated with *phoR*, Rv0081, Rv0600c, *sigB*, *pepD*, *mprB*, *mtrB*, *senX3*, *devR*, and *prrB*.

## Discussion

TB caused by *M*. *tuberculosis* is the major infectious disease worldwide that causes death [24]. The dynamic balance of the inflammatory response induced by *M*. *tuberculosis* infection is crucial for determining the severity of *M*. *tuberculosis*-mediated diseases [25]. Currently, the emergence and progressive spread of multiple resistant strains of *M*. *tuberculosis* have made the prevention and control of TB extremely challenging, making it a global public health and social concern [26]. As the first-line antituberculosis drug, streptomycin mainly acts on the ribosomes of *M*. *tuberculosis* and interferes with protein synthesis [27]. The coding genes of the ribosomal proteins S12 and 16S rRNA were *rpsL* and *rrs*, respectively [28]. In SM-resistant strains, the mutation rate of *rpsL* or *rrs* has reached more than 89%, with the mutation rate of *rpsL* being approximately 63.7% and that of *rrs* being approximately 17.3%. When *rpsL* was mutated, streptomycin affinity to the specific site of 16S rRNA was reduced, streptomycin target was lost, and *M*. *tuberculosis* displayed resistance to streptomycin [29]. The 16S rRNA mutation can interact with the S12 protein to form an easily accessible mutation site [30]. It was believed that the high drug resistance of *M*. *tuberculosis* was related to mutations in *rpsL* and *rrs* [31]. However, the current molecular mechanism of *M*. *tuberculosis* resistance to streptomycin has not been fully elucidated. Therefore, it is crucial to further study the genes, proteins, and signaling pathways related to *M*. *tuberculosis* resistance to streptomycin.

DNA methylation is one kind of an epigenetic modification pattern [32, 33]. Recently, multiple studies have reported that genes modulated by methylation are related to drug-resistant *M*. *tuberculosis* [34–36]. Moreover, N(7)-methylguanosine (m(7)G) methyltransferase can methylate 16S rRNA, causing low resistance of *M*. *tuberculosis* to streptomycin [17]. It has also been reported that methylation can improve streptomycin binding in *M*. *tuberculosis* strains [37]. We further investigated the effect of methylation on gene expression in *M*. *tuberculosis* in the context of streptomycin resistance. We screened 188 differentially methylated genes, including 89 hypermethylated and 99 hypomethylated genes, between the SM-resistant and normal group. Moreover, we discovered that these differentially methylated genes were enriched in 74 pathways, particularly metabolic pathways. Studies have shown that infection by pathogenic microorganisms can induce immune cell activation, accompanied by changes in metabolic pathways [38]. Therefore, it is more certain that these differentially methylated genes are closely related to the metabolic pathways in SM-resistant *M*. *tuberculosis*.

Transcriptomics is an important part of functional genomics and a discipline to study gene transcription and transcriptional regulation in cells at the global level [39]. Currently, microarray technology provides a rapid and effective platform for the large-scale discovery and identification of pathogenic genes [40, 41]. Using the microarray technology, previous studies have explored the profile of *M*. *tuberculosis* transcriptome in different samples, such as lipid-rich dormancy model [42], macrophage infected with *M*. *tuberculosis* [43], and sputum samples from patients with TB [44]. Our study further investigated the changes in transcriptome genes in SM-resistant *M*. *tuberculosis*. Differentially expressed genes, including 263 upregulated (*RVnc0036a*, *Rv0115a*, and *Rv3136A*) and 253 downregulated genes (*Rv2308a*, *Rv0609A*, and *Rv3294c*), were detected in SM-resistant *M*. *tuberculosis*. Moreover, we observed that the 516 differentially expressed genes were mainly enriched in 79 pathways, especially histidine metabolism. Previous studies have shown that histidine metabolism is associated with *M*. *tuberculosis* [45, 46]. Therefore, differential transcriptome genes might provide a theoretical basis for the study of SM-resistant *M*. *tuberculosis*.

Proteome analysis can be used to study the global changes in protein composition or protein abundance [47]. Microbial resistance is a major problem in infection control [48, 49]. The study of the drug resistance mechanisms will improve the effectiveness of current antimicrobial agents [50]. Our study also identified three hub genes (*coaE*, *fadE5*, and *mprA*) based on methylation, transcriptome, and proteome in SM-resistant *M*. *tuberculosis*. *CoaE* plays an important role in the last step of coenzyme A biosynthesis [51]. The overexpression of *coaE* increased doxorubicin production in the doxorubicin-producing wild-type strain [52]. Silencing of *coaE* was bacteriostatic for *M*. *tuberculosis* [53]. Moreover, previous studies have shown that *coaE* is a suitable target for the development of inhibitors against *M*. *tuberculosis* [54, 55]. However, the relationship between *coaE* expression and drug resistance has not been reported. *FadE5* is an acyl-coenzyme A dehydrogenase, which introduces unsaturation to carbon chains in lipid metabolism pathways and is involved in cell wall biosynthesis [56, 57]. The expression of *fadE5* was altered under different antibiotic resistance and virulence [58, 59]. Furthermore, *fadE5* overexpression increased drug resistance to ethambutol and streptomycin in *M*. *smegmatis* [56]. Therefore, we speculated that *fadE5* participates in streptomycin resistance in *M*. *tuberculosis*. *MprA* and *MprB* are two-component signaling systems in *M*. *tuberculosis* that participate in maintaining persistent, latent infections [60, 61]. A previous study indicated that this system can be targeted for developing new anti-mycobacterial agents, particularly against drug-resistant *M*. *tuberculosis* [62]. Overall, *coaE*, *fadE5*, and *mprA* may play an essential role in SM-resistant *M*. *tuberculosis*.

Moreover, the PPI network of the hub genes *coaE*, *fadE5*, and *mprA* was associated with other proteins, providing a clue for further study. However, this study was only a preliminary exploration, and more experiments are needed to confirm the roles of *coaE*, *fadE5*, and *mprA* in SM-resistant *M*. *tuberculosis*. Therefore, in our future studies, *coaE*, *fadE5*, and *mprA* will be targeted for further exploration of the mechanism of SM-resistant *M*. *tuberculosis*. In addition, we will use BSP and MSP technologies to focus on the methylation level of the promoter or transcriptional start site and its regulation mechanism on gene expression. The clinical strains of patients with SM-resistant TB will be collected for further validation.

## Conclusion

We discovered three hub genes (*coaE*, *fadE5*, and *mprA*) through further analysis of the methylation, transcriptome, and proteome of SM-resistant *M*. *tuberculosis*. The protein interaction networks of *coaE*, *fadE5*, and *mprA* were also analyzed. Our study revealed that methylation-related *coaE*, *fadE5*, and *mprA* might contribute significantly to the study of SM-resistant *M*. *tuberculosis*.

## Supporting information

S1 Table Differentially methylated genes between the streptomycin-resistant and normal groups. (XLS)

S2 Table KEGG analysis of differentially methylated genes between the streptomycin-resistant and normal groups. (XLS)

S3 Table Differentially expressed genes between the streptomycin-resistant and normal groups. (XLS)

S4 Table KEGG analysis of differentially expressed genes between the streptomycin-resistant and normal groups. (XLS)

S5 Table Level of differential proteins in the streptomycin-resistant and normal groups. (XLS)

S6 Table Detailed information of *coaE*, *fadE5*, and *mprA* on the methylation, transcriptome, and proteome in streptomycin-resistant *M*. *tuberculosis*. (XLS)

S7 Table Details of interaction information of the three hub genes in Fig 5. (XLS)

## Abbreviations

*coaE*: Dephospho-CoA kinase
*fadE5*: Probable acyl-CoA dehydrogenase *FADE5*
FC: fold change
FDR: false discovery rate
HPLC: high-performance liquid chromatography
iTRAQ: isobaric tags for relative and absolute quantification
LJ: Löwenstein–Jensen
*M*. *Tuberculosis*: *Mycobacterium tuberculosis*
*mprA*: DNA-binding transcriptional repressor
PPI: protein–protein interaction
SM-resistant: Streptomycin-resistant
STRING: Search Tool for the Retrieval of Inter-acting Genes
TB: Tuberculosis
